# A novel strain of *Yarrowia lipolytica* as a platform for value-added product synthesis from glycerol

**DOI:** 10.1186/s13068-016-0593-z

**Published:** 2016-08-30

**Authors:** Aleksandra M. Mirończuk, Dorota A. Rzechonek, Anna Biegalska, Magdalena Rakicka, Adam Dobrowolski

**Affiliations:** Department of Biotechnology and Food Microbiology, Wroclaw University of Environmental and Life Sciences, Chełmońskiego 37, 51-630 Wrocław, Poland

**Keywords:** *Yarrowia lipolytica*, Glycerol, Erythritol, Citric acid, Metabolic engineering

## Abstract

**Background:**

Increasing interest of non-conventional yeasts has been observed for many years due to their biochemical characteristics and potential applications. Well-studied, oleaginous yeast *Y. lipolytica* is an attractive host for converting a low-cost glycerol, into value-added products such as erythritol (sweetener) or citric acid. Glycerol is an important renewable feedstock and is the main co-product of biodiesel production, which is nowadays applied on a large commercial scale. To this end, we engineered the yeast *Y. lipolytica* to increase the productivity of this strain.

**Results:**

In this light, we enhanced glycerol assimilation by over-expression of the *YALI0F00484g* gene encoding glycerol kinase (GK) and gene *YALI0B02948g* encoding glycerol-3-P dehydrogenase (GDH). The modified strains have been tested for glycerol consumption rate and erythritol and citric acid synthesis under various conditions. Here, we show that the overexpression of GK and GDH, increased glycerol consumption resulting in rapid erythritol and citric acid synthesis. Next, we combined the two genes in the tandem gene construct for the simultaneous co-expression of GK and GDH, which further increased the desired product synthesis. The glycerol consumption was explored in a 5-L bioreactor and the engineered strains were able to utilize 150 g/L glycerol within 44–48 hours. The erythritol productivity for GK overexpression and co-expression of GK and DGH was 24 and 35 %, respectively, over the control strain. Moreover, we established conditions for the production of citric acid at pH 3.0, the engineered strains increased citric acid production 14-fold over the control.

**Conclusion:**

This work demonstrates the excellent capacity of the engineered strains as a starting platform for further modification for broad-range value-added product biosynthesis from glycerol. This study presents the highest reported titer citric acid at low pH to date. The process parameters such as productivity and yield of erythritol and citric acid were significantly elevated, what is valuable for industrial applications.

**Electronic supplementary material:**

The online version of this article (doi:10.1186/s13068-016-0593-z) contains supplementary material, which is available to authorized users.

## Background

Modification of microbial metabolism can provide efficient production of value-added chemicals from low-value substrates. In particular, employing microorganisms as producers presents a promising alternative to chemical synthesis of many products widely used in industry such as polyols, organic acids and biofuels. *Yarrowia lipolytica* is one of the most well studied non-conventional yeasts known for its oleaginous properties [1], heterologous protein expression, production of polyols and organic acids [2–6]. Because this yeast is Generally Recognized as Safe (GRAS), it can be easily used in the food and pharmaceutical industries. A unique feature of *Y. lipolytica* is its ability to use unspecific carbon sources such as fatty acids, alkanes or crude glycerol, what is preferred for the production on the industrial scale. For these reasons, a number of studies have been conducted with the goal of enhancing the productivity of this species [7–10]. One of the desired products synthesized by *Y. lipolytica* is erythritol, a natural sweetener, which belongs to the group of polyols. Erythritol is low caloric and possesses non-insulin stimulant properties; it can accordingly be used by diabetics. Moreover, it has been shown that erythritol prevents caries [11] and has the lowest dose–effect among all polyols. Given these advantageous properties, increased demand for this product has been observed. Nowadays, biotechnological production of erythritol based on microbial fermentation is a safe and environmentally friendly process [12]. In yeast, erythritol synthesis occurs during high osmotic pressure; therefore, industrial production of this compound requires a high concentration of glucose (up to 40 %) in the medium, what has an enormous impact on the final product’s market price [13]. Given this fact, it is crucial to find an alternative, low-cost carbon source for erythritol synthesis.

Another industrially important compound produced by *Y. lipolytica* in enormous quantities is citric acid. This organic acid is commercially used as an acidity regulator and flavor enhancer in the food industry, but it is also important in the pharmaceutical and cosmetics industries. On the commercial scale, citric acid is largely produced by the mycelial fungus *Aspergillus niger*, but in the past few years many studies have been focused on the potential use of the yeast *Y. lipolytica* [14, 15].

**Fig. 1:**
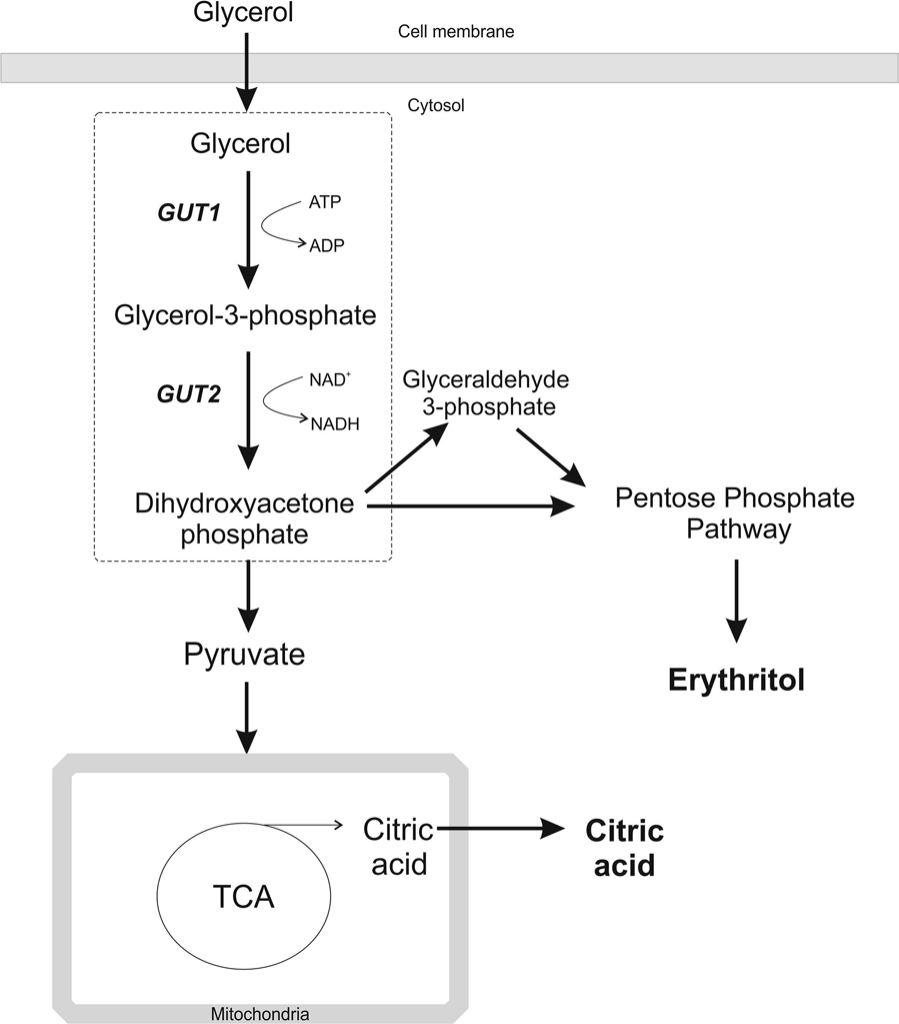
Overview of the principal metabolic pathways for erythritol and CA production in *Y. lipolytica*. In *Y. lipolytica*, glycerol is assimilated via phosphorylation by a glycerol kinase and subsequently is dehydrogenased to dihydroxyacetone phosphate by glycerol-3-P dehydrogenase. Then, the erythritol is synthetized via a pentose phosphate pathway in response to high osmotic stress. Citric acid synthesis occurs in mitochondria in the TCA cycle

The primary limitations faced by the researchers involved in biotechnological processes are cost, enhancing productivity and boosting the yield of the desired product. To address these issues, an alternative source of carbon has been tested for industrial production. One of the suitable low-cost substrates for *Y. lipolytica* is glycerol, the primary by-product of biodiesel production, which is nowadays produced on an enormous commercial scale. The ever-growing world population necessitates progressively more energy sources. Therefore, the production of biodiesel increases. Moreover, glycerol is produced by several other industries, such as fat saponification and stearin production. It is worth noting that despite the high contamination, crude glycerol is easily utilized by the yeast *Y. lipolytica* [16]. In *Y. lipolytica*, glycerol is assimilated by phosphorylation pathway, and the substrate is first phosphorylated to 3-P-glycerol by a glycerol kinase (GK) and subsequently is dehydrogenated to dihydroxyacetone phosphate by glycerol-3-P dehydrogenase (GDH) (Fig. 1).

To enhance the assimilation of glycerol, we engineered the metabolism of *Y. lipolytica* and overexpressed the first two genes involved in this process, namely GK encoded by *GUT1* and GDH encoded by *GUT2*. Next, we coupled the overexpression of *GUT1* and *GUT2* with *Y. lipolytica*’s capacity to grow at low pH levels. By employing metabolic modification and process optimization, we are able to improve citric acid titer to achieve 63.9 g/L at pH 3.0 and 93 g/L at pH 6.0. Moreover, we demonstrated erythritol synthesis 35 % higher than that of the control in the engineered strain. Our results reveal that coupling the overexpression of *GUT1* and *GUT2* enables effective glycerol assimilation toward the synthesis of desired products.

## Methods

### Microorganisms, media and culture conditions

The *Y. lipolytica* strains used in this study were derived from the wild-type *Y. lipolytica* A101 [17]. All the strains used in this study are listed in Table 1.

**Table 1:** Strains and plasmids used in this study

Strain	Genotype or plasmid	Source
*E. coli*		
DH5α	F− endA1 glnV44 thi-1 recA1 relA1 gyrA96 deoR nupG Φ80dlacZΔM15 Δ(lacZYA-argF)U169, hsdR17(rK− mK+), λ−	[35]
DH5α	pMCSUAS1B16-TEF-lacZ	[23]
DH5α	pMT-Ura-Gut-XT	[9]
DH5α	pADUTGut1	This study
DH5α	pADUTGut2	This study
*Y. lipolytica*		
A101	Wild type	[17]
AJD	*MATA*, A101: ura3-302	[9]
AJD pADUTGut1	*MATA*, A101: ura3-302, pADUTGut1	This study
AJD pADUTGut2	*MATA*, A101: ura3-302, pADUTGut2	This study
AJD pADUTGut1/2	*MATA*, A101: ura3-302, pADUTGut1 pADUTGut2	This study

*Escherichia coli* strains were cultivated in LB (BTL, Poland) medium according to standard protocols [18]. Rich Yeast Extract Peptone Glucose (YPD) medium was used for the yeast inoculum preparation and contained in 1 % (w/v) yeast extract (Merk, Germany), 1 % (w/v) peptone (Biocorp, Poland) and 2 % (w/v) glucose (Merk, Germany).

For the shake flask experiment, we prepared the media as follows: Glycerol Assimilation Medium: YNB medium without amino acids (Sigma Aldrich, Germany) supplemented with 100 g/L pure glycerol (Chempur, Poland), pH 6.0. Erythritol Fermentation Medium (g/L): 100 glycerol (Chempur), 2.3 (NH_4_)_2_SO_4_ (Chempur), 1 MgSO_4_ × 7H_2_O (Chempur), 0.23 KH_2_PO_4_, NaCl 26.4 (Chempur), 1 yeast extract (Merk, Germany) and 3 CaCO_3_ (Chempur), pH 3.0. Citric acid production in CA Fermentation Medium (g/L): 100 glycerol, 2.7 (NH_4_)_2_SO_4_, 1 MgSO_4_ × 7H_2_O, 0.22 KH_2_PO_4_, 1.6 yeast extract and 3 CaCO_3_, pH 3.0. The pH of the media was adjusted by addition of 40 % NaOH or 20 % HCl. The cultures were performed in three replicates.

### Bioreactor studies

To prepare an inoculation culture for fermentation in a bioreactor, the cultures were grown in 0.3-L flasks (containing 0.1 L of YPD medium) on a shaker at 28 °C for 72 hours at 140 rpm. Glycerol Assimilation Medium was prepared as follows the YNB medium (without amino acids) was prepared according to the manufacturer’s instructions (Sigma Aldrich), supplemented with 150 g/L of 98 % (wt/wt) pure glycerol (Chempur), pH 6.0.

Erythritol production was conducted in a medium (Erythritol Fermentation Medium) consisting of (g/L): 150 glycerol, (Chempur), 2.3 (NH_4_)_2_SO_4_ (Chempur), 1 MgSO_4_ × 7H_2_O (Chempur), 0.23 KH_2_PO_4_, NaCl 26.4 (Chempur), 1 yeast extract (Merk, Germany), pH 3.0.

Citric acid production was carried out using the following medium (CA Fermentation Medium) (g/L): 150 glycerol, 2.7 (NH_4_)_2_SO_4_, 1 MgSO_4_ × 7H_2_O, 0.23 KH_2_PO_4_ and 1.6 yeast extract, pH 3.0.

An inoculum of 0.2 L was introduced into the bioreactor containing the production medium. The cultivations were performed in a 5-L jar bioreactor (Biostat B Plus, Sartorius, Germany) with a working volume of 2 L at 28 °C. The aeration was fixed at 1 L/min. The stirrer speed was adjusted to 800 rpm. The pH was maintained automatically at 3.0 or 6.0 via the addition of NaOH (40 % w/v). The amount of the supplied NaOH has been taken into account during calculations of the metabolite concentrations. To limit, evaporation during the batch cultures, the exhaust gases passed into the exhaust condenser in which the moisture was removed and returned to the vessel. The cultures were performed in three biological replicates.

### Cloning and transformation protocols

All restriction enzymes were purchased from FastDigest Thermo Scientific™ (USA) and all the digestions were performed according to standard protocols. The PCR was set up using recommended conditions and Phusion high-fidelity DNA polymerase (Thermo Scientific™). The ligation reactions were performed for 10 minutes at room temperature using T4 DNA Ligase (Thermo Scientific™). The gel extractions were performed using the Gel Out gel extraction kit purchased from A&A Biotechnology (Poland). The *E. coli* minipreps were performed using the Plasmid Mini Kit (A&A Biotechnology). Transformation of *E. coli* strains was performed using standard chemical protocols [18]. Genomic DNA (gDNA) was extracted from *Y. lipolytica* using the Genomic Mini AX Yeast Spin kit (A&A Biotechnology, Poland). The obtained plasmids were digested with MssI to create linear expression cassettes devoid of *E. coli* DNA and surrounded by *Y. lipolytica* rDNA for targeted integrations. First, *Y. lipolytica* AJD [9] was transformed with *GUT1* or *GUT2* overexpression cassette, according to the lithium acetate method described before [19], resulting in strains AJD pADUTGut1 or AJD pADUTGut2, respectively. The transformants were plated out on selective media [9] and were confirmed via gDNA extraction and three distinct PCR confirmations. Next, auxotrophies were restored via excision using the Cre-lox recombinase system following transformation with the replicative plasmid pUB4-Cre1(JME547) [20]. Consequently, strain AJD pADUTGut1 was transformed with *GUT2* overexpressing cassette, resulting in strain AJD pADUTGut1/2.

### Construction of overexpression plasmids

The UAS1B_16_-TEF promoter was gel-extracted from the plasmid pMCSUAS1B_16_-TEF- lacZ [23] with a Bsp119I (BstBI) and AscI. This was inserted into adequate sites of pMT-Ura-Gut-XT [9] to form the plasmid pADUTGut1.

After amplification of *Y. lipolytica* DNA with the primers Gut2-AscI-F (5′GTACGGCGCGCCATGTTCAGAACCATTCGAAAAC-3′) and Gut2-NheI-R (5′-GTACGCTAGCTTATTTGTCCTTGGGGGTAAG-3′), the 1861-bp PCR fragment was digested with AscI and NheI and cloned into the corresponding sites of gel-extracted pADUTGut1 to yield pADUTGut2.

### RNA isolation and transcript quantification

The shake flask cultures were grown for 48 hours in YNB medium supplemented with glycerol (100 g/L). Next, the cultures were collected and centrifuged for 5 minutes at 12,000*g*. The RNA was extracted using a Total RNA Mini Plus kit (A&A Biotechnology, Poland). Each sample was treated with DNAse I (ThermoScientific™) according to the manufacturer’s instructions. We measured RNA quantities using a Biochrom WPA Biowave II spectrophotometer (Biochrom Ltd., UK) equipped with a TrayCell (Hellma Analytics, Germany), and the samples were stored in a −80 °C freezer. We conducted cDNA synthesis using Maxima First Strand cDNA. Synthesis kits for RT-qPCR (ThermoScientific™) were used according to the manufacturer’s instructions. We carried out qRT-PCR analyses using a DyNAmo Flash SYBR Green qPCR Kit (ThermoScientific™) and the Eco Real-Time PCR System (Illumina, USA). Primers for RT-PCR were designed as follows: gene (*GUT1*, *YALI0F00484g*) encoding the glycerol kinase and gene (*GUT2*, *YALI0B02948g*) encoding the glycerol-3-P dehydrogenase were used as a templates. Primers qGUT1-F (5′-GTACCACCTCCACCCGTTTC-3′) and qGUT1-R (5′-CACCTTGATGCCCTGGGTTC-3′) bind at 32 bp and at 219 bp of the *GUT1* gene, respectively, resulting in 188 bp PCR product. Next, gene *YALI0B02948g* encoding glycerol-3-P dehydrogenase possesses one intron (664 bp), primer qGUT2-F (5′-GGTCGCCGTTGTTGGTTCTG-3′) binds in the first exon at 135 bp, and primer qGUT2-R (5′-CTCGAACCTCGGGCTCAAAG-3′) binds in the second exon at 826 bp. The obtained PCR product in qRT-PCR is 101 bp. The results were normalized to actin gene (ACT-F 5′-GAGTCACCGGTATCGTTC-3, ACT-R 5′-GCGGAGTTGGTGAAAGAG-3′) and analyzed using the ddCT method [21]. Samples were analyzed in triplicate.

### Bioscreen C

The inoculation cultures were grown for 24 hours in YPD medium. Next, we centrifuged the overnight cultures and washed them with sterile water. Next, the yeast strains were grown in 100-well plates in 150 μL of YNB medium supplemented with glycerol 5 % (v/v) or glucose 2 % (w/v). The OD_600_ of the cells was standardized to 0.15. Quintuple experiments were performed at 28 °C under constant agitation with a Bioscreen C (Oy Growth Curves Ab Ltd., Finland). Growth was monitored by measuring the optical density (OD) at 420–560 nm every 30 minutes for 48 hours.

### Analytical methods

Samples (10 mL) from the batch cultures were centrifuged for 10 minutes at 4 °C and 5500*g* and harvested via filtration on 0.45-μm pore membranes and washed twice with distilled water. The biomass was determined gravimetrically after drying at 105 °C. The concentrations of glycerol, erythritol, arabitol, mannitol and CA were determined with HPLC using a HyperRez Carbohydrate H+ Column (Thermo Scientific, Waltham, MA) coupled to a UV (*λ* = 210 nm) (Dionex, Sunnyvale, USA) and a refractive index detector (Shodex, Ogimachi, Japan). The column was eluted with 25 mM of trifluoroacetic acid at 65 °C and a flow rate of 0.6 mL min^−1^.

The diagnostic kits D-isocitric acid (Boehringer Mannheim, Germany) were used for the assay isocitric acid concentrations.

### Calculation of fermentation parameters

To take into account the medium dilution due to the addition of the NaOH required for pH control, the amounts of erythritol, citric acid and the by-products in the culture broth were used to calculate the mass yield of erythritol, citric acid (*Y*_ERY_, *Y*_CA_) and the volumetric erythritol, citric acid productivity (*Q*_ERY_, *Q*_CA_). The mass yield of erythritol and citric acid (*Y*_ERY_, *Y*_CA_) was expressed in g/g from glycerol and was calculated according to the equation:

$$Y_{\text{ERY}} = \, P/S.$$

The volumetric erythritol (*Q*_ERY_) and CA (*Q*_CA_) productivities expressed in g/L/h were calculated using the equation:

$$Q \, = \, P/ \, V \cdot \, t,$$

where *P* is the amount of product in the culture liquid at the end of cultivation (g), *S* is the total amount of glycerol consumed (g), *V* is the initial volume of culture liquid (l) and *t* is the fermentation time (h).

## Results and discussion

### Overexpression of the *GUT1* and *GUT2* genes in *Y. lipolytica*

**Fig. 2:**
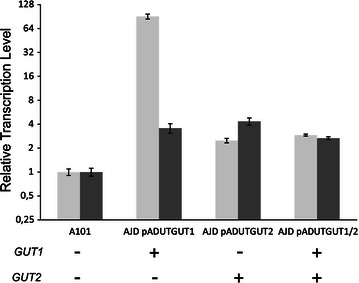
Analysis of strains overexpressing *GUT1* (*light gray bars*) and/or *GUT2* (*dark gray bars*). Relative quantification of RNA transcript using RT-PCR, actin was used as the reference gene. The analysis was performed in triplicate, and the standard errors were estimated using Illumina Eco software

*GUT1* and *GUT2* are two genes encoding the first two enzymes that are involved in glycerol assimilation into the cell of *Y. lipolytica* [22]. Therefore, to increase glycerol assimilation by yeast cells, we overexpressed these genes separately and in tandem to verify which combination was the most efficient. In this study, we used a hybrid promoter containing 16 upstream activating sequences enhancing the expression of the *TEF* promoter [23]. Proper integration into the genome was verified by PCR (see Additional file 1), and we also checked the overexpression via RT-PCR of the total RNA. According to our assumptions, all the engineered strains exhibited elevated expression of *GUT1* and *GUT2* (Fig. 2). Surprisingly, the expression level of *GUT1* gene was significantly higher when it was singly overexpressed rather than co-expressed with *GUT2.* A similar effect in co-expression was observed before (Tai and Stephanopoulos [10]) when only one showed very high expression during the co-expression of two genes. In our study, a slight upregulation of *GUT2* was observed in the AJD pADUTGut1 and for *GUT1* in AJD pADUTGut2 strain. This effect was caused by the regulatory relationship in place between these genes. Given these results, we tested the effect of *GUT1* and *GUT2* overexpression on the efficiency of glycerol assimilation.

**Fig. 3:**
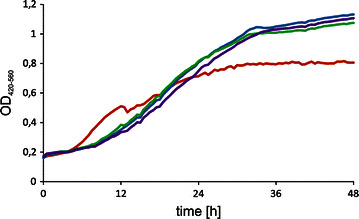
Growth curves of various *Y. lipolytica* strains: A101 (*red line*), AJD UTGut1 (*blue line*), AJD UTGut2 (*green line*) and AJD UTGut1/2 (*purple line*). The strains were grown on a YNB/glycerol medium. Quintuple experiments were performed at 28 °C under constant agitation using a Bioscreen C (Oy Growth Curves Ab Ltd.)

We sought to verify the growth difference between the modified strains and the wild type on YNB medium supplemented with glycerol. As a control medium YNB with glucose was used. As in Fig. 3, all the engineered strains showed extended lag phase. However, after 20 hours of growth, rapid growth was noted for these strains. Maximum growth was observed after 24 hours, a finding that is in agreement with the activity maximum of the *TEF* promoter [23]. Later, the *GUT1*- and/or *GUT2*-overexpressing strains achieved a higher OD compared with that of the control. Strain A101 grew easily on glycerol. In agreement with a previous study [24], we did not observe any difference in growth on glucose between the strains (data not shown).

### Overexpression of *GUT1* and *GUT2* leads to significant increases in metabolite production from glycerol

Next, we performed a shake flask experiment given three varied conditions. In the first case, the strains were grown in Glycerol Assimilation Medium (pH 6.0). In the second case, the strains were grown in Erythritol Fermentation Medium (pH 3.0). In the third case, the strains were grown in CA Fermentation Medium at pH 3.0 (for additional details, see the “Methods”).

**Fig. 4:**
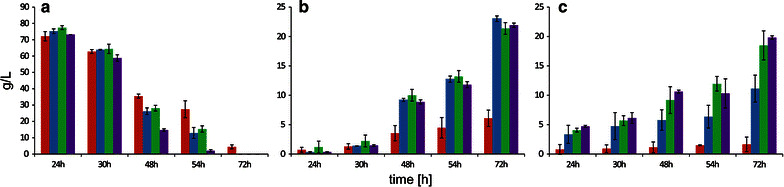
Glycerol assimilation in Glycerol Assimilation Medium, pH 6.0 (**a**), erythritol synthesis in Erythritol Fermentation Medium (**b**) and citric acid synthesis in CA Fermentation Medium (**c**) at pH 3.0 in a shake flask experiment using the *Y. lipolytica* strains A101 (*red*), AJD UTGut1 (*blue*), AJD UTGut2 (*green*) and AJD UTGut1/2 (*purple*). The cultures were performed in three biological replicates. The *error bars* represent the standard deviation

Previous experiments have shown that the strains carrying overexpression cassettes *GUT1* and/or *GUT2* assimilated glycerol more efficiently than the wild type and exhibited better growth-rate profiles. To address the question, if rapid glycerol utilization would be observed at enhanced scale and if it would be associated with enhanced metabolite production, we performed shake flask experiments. In the first series of studies, the strains were cultivated in YNB with an initial glycerol concentration adjusted at 100 g/L. In agreement with previous experiment, all the modified strains depleted glycerol more rapidly than the control. Figure 4 and Table 2 summarize the results of the shake flask experiments. Interestingly, after 24 hours, the control strain utilized the largest amount of glycerol. However, after next 6 hours the AJD pADUTGut1/2 strain used more substrate. Within the next 24 hours, all the engineered strains consumed more glycerol than A101. *Y. lipolytica* is known to produce CA at pH 6.0 [25], and we also observed this phenomenon. Strikingly, A101 produced 2.0 g/L, AJD pADUTGut1 2.5 g/L, AJD pADUTGut2 2.7 g/L and AJD pADUTGut1/2 2.95 g/L of citric acid. These findings revealed that efficient glycerol assimilation leads to elevated synthesis of this metabolite under the described conditions.

**Table 2:** The parameters of the processes conducted in flask and batch cultivation

	A101	AJD pADUTGut1	AJD pADUTGut2	AJD pADUTGut1/2
Shake flaks experiment				
Glycerol Assimilation Medium (pH 6.0)				
Glol_cons_ (g/L)^a^	72.57 ± 5.18	87.08 ± 3.32	84.64 ± 1.94	97.92 ± 0.67
Erythritol Fermentation Medium (pH 3.0)				
* Q*_ERY_ (g/L/h)	0.08 ± 0.01	0.32 ± 0.01	0.30 ± 0.04	0.30 ± 0.01
* Y*_ERY_ (g/g)	0.06 ± 0.01	0.23 ± 0.01	0.21 ± 0.03	0.22 ± 0.01
CA Fermentation Medium (pH3.0)				
* Q*_CA_ (g/L/h)	0.02 ± 0.01	0.15 ± 0.01	0.26 ± 0.01	0.28 ± 0.01
* Y*_CA_ (g/g)	0.02 ± 0.01	0.11 ± 0.01	0.18 ± 0.01	0.20 ± 0.01
Batch fermentation				
Glycerol Assimilation Medium (pH 6.0)				
Glol_cons_ (g/L)^b^	142.43 ± 3.86	149.58 ± 0.50	150 ± 0.01	150 ± 0.01
Erythritol Fermentation Medium (pH 3.0)				
* Q*_ERY_ (g/L/h)	0.80 ± 0.03	0.99 ± 0.02	0.58 ± 0.01	1.08 ± 0.02
* Y*_ERY_ (g/g)	0.38 ± 0.01	0.48 ± 0.01	0.28 ± 0.02	0.52 ± 0.01
CA Fermentation Medium (pH3.0)				
* Q*_CA_ (g/L/h)	0.05 ± 0.01	0.63 ± 0.01	0.57 ± 0.01	0.69 ± 0.01
* Y*_CA_ (g/g)	0.03 ± 0.01	0.40 ± 0.01	0.36 ± 0.04	0.43 ± 0.01

^a^ *Glol*_*cons*_ glycerol consumed at 54 hours of the process

^b^ *Glol*_*cons*_ *(g/L)* glycerol consumed at 48 hours of the process

*Q*_*ERY*_ the erythritol productivity, *Y*_*ERY*_ the erythritol yield, *Q*_*CA*_ the citric acid productivity, *Y*_*ERY*_ the citric acid yield

The analysis was performed in triplicate. Cultures condition was described in the “Methods”

Given these results, we sought to increase the erythritol titer by applying high osmotic conditions. This approach was based on prior experiments that showed that increasing osmotic stress increased erythritol synthesis [26, 27]. Indeed, as seen in Fig. 4b, the overexpression of *GUT1* and/or *GUT2* increased the erythritol titer nearly four-fold over the control after 72 hours. The A101 strain produced only 6.07 g/L; AJD pADUTGut1 produced 23.02 g/L, AJD pADUTGut2 produced 21.4 g/L and AJD pADUTGut1/2 produced 21.95 g/L. It is worth noting that all modified strains were able to completely deplete glycerol in Erythritol Fermentation Medium within 72 hours (data not shown). Moreover, the process parameters were significantly increased, the erythritol productivity (*Q*_ERY_) and yield (*Y*_ERY_) in all the engineered strains exceeded those of the control strain by a factor of four (Table 2).

Since erythritol is produced by cells in response to high osmotic pressure, inhibiting the production of erythritol requires decreasing the osmotic stress by reducing the salinity of medium. Due to this modification, the osmotic pressure was reduced from 2.05 Osmol/kg in the Erythritol Fermentation Medium to 1.2 Osmol/kg in the CA Fermentation Medium; the pH of the medium remained at 3.0. It is known that the optimal pH for CA production via *Y. lipolytica* ranges from 5.0 to 6.0 [6, 25], and it decreases at lower pH. In agreement with previous studies, the same effect was observed for wild type A101, which produced only 1.64 g/L CA at pH 3.0 (Fig. 4c). Interestingly, the single overexpression of *GUT1* resulted in nearly a seven-fold increase in CA production compared with that of the control. Furthermore, the overexpression of *GUT2* leads to high synthesis ranged 18.49 g/L, which is eleven-fold over that of the control. This effect results from the high carbon flux through pyruvate and consequently in high citrate production in the mitochondria (Fig. 1). The coexpression of *GUT1* and *GUT2* enables increased throughput, and the synergic overexpression of these two genes leads to 19.82 g/L of CA, which is twelve-fold over that of the control strain. Moreover, coupling *GUT1* and *GUT2* overexpression with low osmotic pressure yields an enormous CA titer at low pH. Strikingly, it has been shown that metabolic engineering can improve product titer and yields but results in a decrease in cell growth [10, 28]. However, we did not observe this effect. The biomass yields remained constant regardless of the strain that we used at pH 3.0; they oscillated between 11.8 and 14.1 g/L. In the medium at pH 6.0, they ranged between 20.45 and 20.95 g/L.

The most interesting outcome of these experiments was the improvement in the process parameters, namely the glycerol consumption rate, the productivity and the yield. Table 2 summarizes the parameters of the shake flask experiments. All the engineered strains assimilated glycerol more rapidly than the wild type. After 54 hours of cultivation, the lowest rate of glycerol consumption (Glol_cons_) was noted for the control (72.57 g/L). The highest Glol_cons_ was achieved by AJD pADUTGut1/2, and this strain assimilated 97.92 g/L of glycerol or an increase of 26 % over that of the control (Table 2). All of the engineered strains outperformed the control and consumed all the glycerol within 60 hours of cultivation; wild type required 74 hours. This result reveals that the overexpression of *GUT1* and *GUT2* enhances glycerol utilization. Shortening the time of the process is desirable in industry to reduce production costs.

Furthermore, the parameters of erythritol production were significantly improved. The erythritol productivity (*Q*_ERY_) and the yield (*Y*_ERY_) observed for the control were both very low: 0.08 g/L/h and 0.06 g/g, respectively. The expression of *GUT1*, *GUT2* or both of these genes resulted in *Q*_ERY_ values ranging from 0.30 to 0.32 g/L/h and *Y*_ERY_ values ranging from 0.21 to 0.23 g/g, which is a significant improvement. The most noteworthy enhancement was observed for CA production. As noted above, the yeast *Y. lipolytica* produces low quantities of CA at low pH; the control exhibited a CA productivity (*Q*_CA_) and yield (*Y*_CA_) of 0.02 g/L/h and 0.02 g/g, respectively. The expression of *GUT1* resulted in an elevated productivity (0.15 g/L/h) and yield (0.11 g/g), which corresponds to a six-fold increase over the control values. The impact of *GUT2* overexpression on CA synthesis was even more pronounced. The single overexpression led to a *Q*_CA_ of 0.26 g/L/h and a *Y*_CA_ of 0.18 g/g, and the tandem expression of the two genes resulted in a fourteen-fold *Q*_CA_ improvement over the control and a ten-fold *Y*_CA_ improvement over the control (Table 2). Given these results, we further studied the production of erythritol and CA in the bioreactor to scale up the process.

### Fermentation performance in the bioreactor of the *GUT1* and *GUT2* transformants

**Fig. 5:**
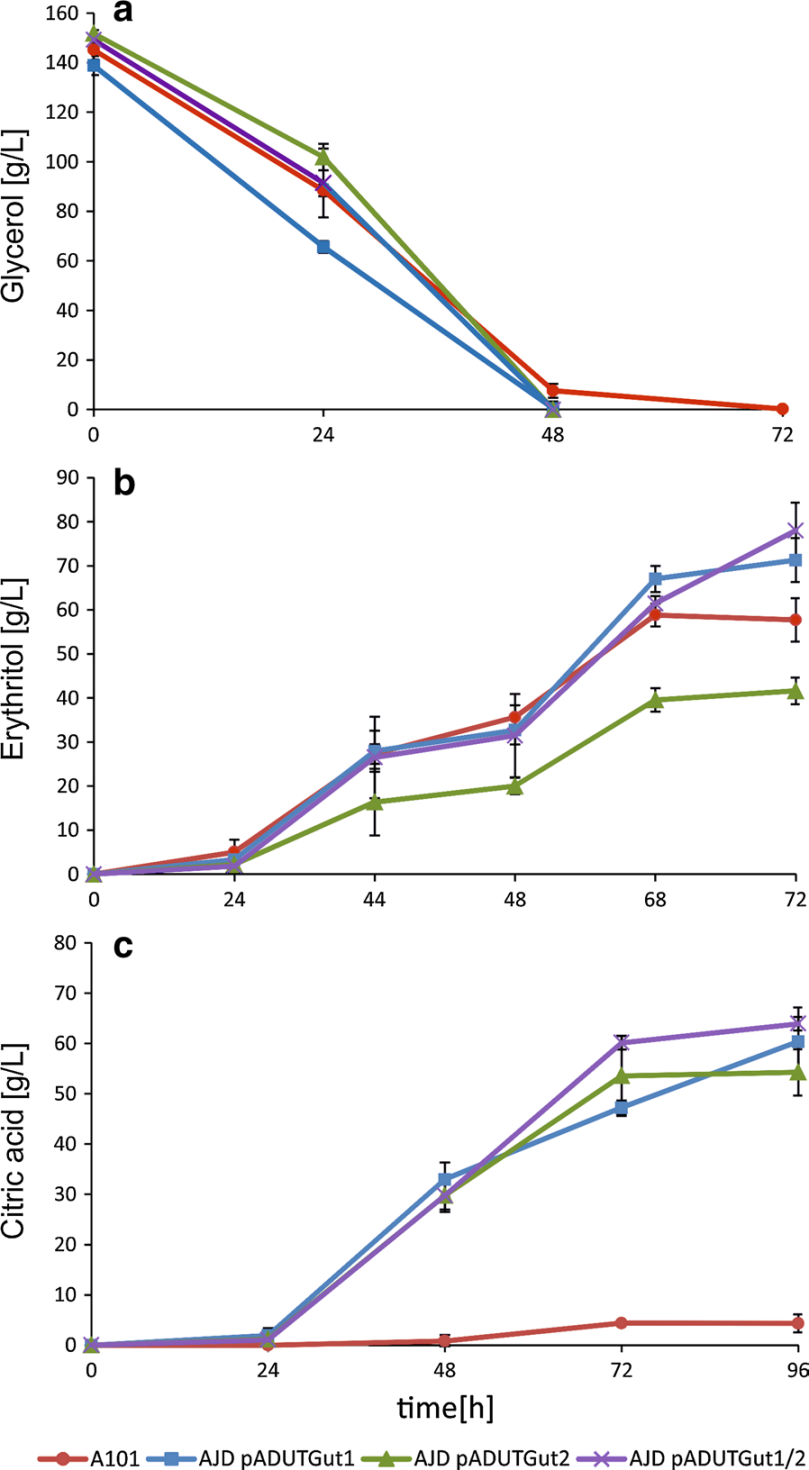
Batch bioreactor fermentations with strains overexpressing GUT1 and/or GUT2, with strain A101 used as a control. The strains were grown in Glycerol Assimilation Medium, pH 6.0 (**a**), in Erythritol Fermentation Medium, pH 3.0 (**b**) or in CA Fermentation Medium, pH 3.0 (**c**). The cultures were performed in three biological replicates

To further characterize the engineered strains and explore their production abilities, we performed large-scale fermentation using a 5-L stirred-tank bioreactor. First, the strains were tested for glycerol assimilation rate in Glycerol Assimilation Medium. Table 3 summarizes the results of the experiments. Again, we used strain A101 as a control, which consumed 150 g/L of glycerol within 72 hours (Fig. 5a). The strain quickly grew to a biomass titer of 18 g/L within 24 hours and to 31.4 g/L afterwards. The CA productivity (*Q*_CA_) reached a value of 0.75 g/L/h and the yield (*Y*_CA_) reached a value of 0.36 g/g. Over the course of the process, A101 produced 53.7 g/L of CA.

**Table 3:** Glycerol utilization by various *Y. lipolytica* strains in Glycerol Assimilation Medium (pH 6.0)

Strain	Time (h)	Biomass (g/L)	Glycerol (g/L)	Erythritol (g/L)	Arabitol (g/L)	Mannitol (g/L)	Citric acid (g/L)	α-Ketoglutaric acid (g/L)
A101	24	18 ± 0.28	88.4 ± 3.8	2.6 ± 3.0	0.3 ± 0.1	1.2 ± 0.8	6.1 ± 1.7	0.5 ± 0.1
	48	31.4 ± 3.7	7.9 ± 2.4	0.5 ± 0.7	0.3 ± 0.2	2.3 ± 0.7	50.3 ± 3.9	0.5 ± 0.1
	72	29.6 ± 2.4	0.2 ± 0.1	0	0.1 ± 0.1	0.3 ± 0.5	53.7 ± 3.5	0.4 ± 0.1
AJD pADUTGut1	24	30.5 ± 2.4	65.6 ± 2.4	4.8 ± 3.0	0.7 ± 0.1	2.5 ± 0.9	9.2 ± 1.7	0.5 ± 0.1
	48	28.6 ± 4.7	0.4 ± 2.4	0	0.5 ± 0.3	3.1 ± 0.7	46.5 ± 3.9	0.5 ± 0.1
	72	29.9 ± 4.0	0	0	0.1 ± 0.1	0.1 ± 0.01	76.9 ± 3.5	0
AJD pADUTGut2	24	21.5 ± 1.3	101.9 ± 5.4	6.0 ± 2.2	1.9 ± 0.2	0.9 ± 0.1	7.2 ± 2.1	1.1 ± 0.1
	48	32.5 ± 2.2	0	4.4 ± 3.3	4.2 ± 0.4	4.8 ± 1.8	70.4 ± 3.2	1.2 ± 0.7
	72	29.5 ± 3.6	0	0	0	0	83.1 ± 0.2	0
AJD pADUTGut1/2	24	24.9 ± 4.6	91.4 ± 13.4	6.8 ± 3.9	1.8 ± 1.9	2.4 ± 1.4	13.1 ± 5.1	0.9 ± 0.7
	48	33.3 ± 1.1	0	1.9 ± 2.4	2.0 ± 2.1	5.6 ± 1.5	70.5 ± 1.1	1.1 ± 0.1
	72	25.8 ± 3.3	0	0	0	0	92.9 ± 10.3	0

For strain AJD pADUTGut1, the glycerol was fully assimilated within 48 hours (Fig. 5a), with a final biomass concentration of 30 g/L. It is noteworthy that the CA content was 76.9 g/L over the course of the fermentation, which corresponds to an over 40 % increase in the titer compared with the control. This resulted in a *Q*_CA_ of 1.07 g/L/h and a *Y*_CA_ of 0.51 g/g. The strain overexpressing *GUT2* consumed 150 g/L of glycerol within 48 h of the process, and its biomass attained 32.5 g/L. The strain produced 83 g/L of CA, with an increased *Q*_CA_ of 1.15 g/L/h and a *Y*_CA_ of 0.55 g/g. The highest titer of CA was observed for the strain co-overexpressing *GUT1* and *GUT2*, and the final CA content was 93 g/L with *Q*_CA_ 1.29 g/L/h and *Y*_CA_ 0.62 g/g. Interestingly, after the complete depletion of glycerol, the engineered strains utilized side metabolites such as erythritol, mannitol and arabitol (Table 3). As a result, the carbon flux was towards CA synthesis. It is worthwhile noting that we did not observe a decrease in the biomass production, which was observed previously for modified *Y. lipolytica* strains [10, 28]. On the contrary, all the modified strains produced more biomass within the first 24 hours of the course, and the wild type achieved the same biomass level afterward. Many studies have been conducted on CA production by *Y. lipolytica* at pH 5.0–6.0 [15, 29], but in these reports, the strains obtained lower productivities of 0.52–0.85 g/L/h and *Y*_CA_ 0.25–0.53 g/g [15].

### Erythritol synthesis by the *GUT1* and *GUT2* transformants

Next, we investigated erythritol production using engineered strains. Tables 2 and 3 summarize the results of this study. It has been reported that different strains of *Y. lipolytica* possess different abilities in terms of erythritol synthesis [26]. Strain A101 has a low ability in terms of erythritol synthesis [15]. To force higher carbon flux toward erythritol synthesis, we introduced high osmotic stress, as was described previously in the literature [26].

In this experiment, the control strain produced 57.7 g/L of erythritol (Fig. 5b), with *Q*_ERY_ 0.80 g/L/h and *Y*_ERY_ 0.38 g/g. Moreover, due to stress conditions, glycerol was not completely depleted within 72 hours. Additionally, the biomass and CA synthesis were reduced compared with the data obtained from the Glycerol Assimilation Medium. The strain overexpressing *GUT1* produced 71.3 g/L of erythritol (Fig. 5b), and *Q*_ERY_ and *Y*_ERY_ were enhanced to 0.99 g/L/h and 0.48 g/g, respectively. Again, the biomass and CA synthesis were reduced, a finding that is of great value because the carbon flux was redirected towards the erythritol production. Surprisingly, the strain overexpressing *GUT2* yielded high quantities of CA and achieved a half titer of erythritol (Table 4). This result suggests that *GUT2* is crucial for CA synthesis since the carbon flux is redirected towards the TCA cycle. It has been shown that the deletion of *GUT2* leads to increased lipid production [30] because excess carbon flux accumulates as CA (dispersed throughout the supernatant) before reabsorption and is incorporated into the biomass. However, it has been suggested that a re-engineered metabolism enables to directly incorporate carbon into elongating fatty acids, potentially exclusively intracellular metabolites such as acetyl-CoA or malonyl-CoA [31]. Therefore, the role of *GUT2* in CA synthesis is not fully understood.

**Table 4:** Erythritol and by-product synthesis by various *Y. lipolytica* strains in Erythritol Fermentation Medium (pH 3.0)

Strain	Time (h)	Biomass (g/L)	Glycerol (g/L)	Erythritol (g/L)	Arabitol (g/L)	Mannitol (g/L)	Citric acid (g/L)	α-Ketoglutaric acid (g/L)
A101	24	11.3 ± 2.9	122.5 ± 3.4	5.0 ± 2.8	0	0	0	0.3 ± 0.2
	48	19.1 ± 2.2	64.3 ± 6.7	35.6 ± 2.2	0.4 ± 0.2	2.4 ± 1.5	0.4 ± 0.1	0.9 ± 0.2
	72	24.4 ± 1.7	4.5 ± 0.4	57.7 ± 2.7	0	4.1 ± 0.9	1.4 ± 0.2	1.7 ± 0.7
AJD pADUTGut1	24	10.7 ± 4.0	113.5 ± 15.4	3.3 ± 2	0.1 ± 0.1	0.1 ± 0.1	0	0
	48	19.8 ± 3.5	56.8 ± 8.2	32.7 ± 3.3	0.4 ± 0.2	2.1 ± 1.2	0.6 ± 0.2	1.2 ± 0.3
	72	21.6 ± 5.4	6.1 ± 5.6	71.3 ± 5.0	0.6 ± 0.4	5.2 ± 2.0	4.2 ± 1.9	0
AJD pADUTGut2	24	5.8 ± 1.7	130.9 ± 13.4	2.2 ± 1.1	0	0.7 ± 0.3	2.6 ± 1.3	1.0 ± 0.1
	48	16 ± 0.6	78.6 ± 16.2	20.0 ± 1.9	0.8 ± 0.2	0.9 ± 0.1	3.8 ± 1.9	1.2 ± 0.1
	72	19.0 ± 1.4	4.9 ± 1.9	41.6 ± 3.0	1.5 ± 1.0	3.7 ± 1.5	22.8 ± 2.8	0
AJD pADUTGut1/2	24	6.2 ± 1.5	130.1 ± 13.8	1.8 ± 0.2	0	0	1.0 ± 0.1	1.4 ± 0.2
	48	16 ± 2.2	94.8 ± 6.2	31.5 ± 5.5	0.2 ± 0.3	1.6 ± 0.9	1.4 ± 0.32	2.2 ± 1.3
	72	17.5 ± 1.3	7.7 ± 3.6	78.0 ± 6.3	0.5 ± 0.3	4.5 ± 3.5	2.8 ± 0.9	4.4 ± 0.9

**Fig. 6:**
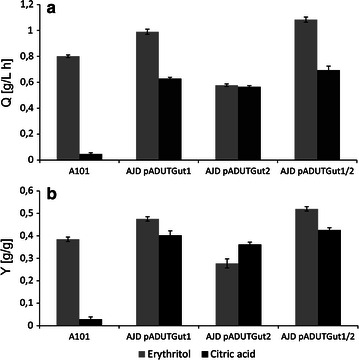
The process parameters of the bioreactor fermentations. The productivity (**a**) and yield (**b**) of the strains overexpressing *GUT1* and/or *GUT2* in Erythritol Fermentation Medium (*gray*) or in CA Fermentation Medium (*black*); we used A101 as a control strain

We further tested the engineered *GUT1/GUT2* strain on erythritol production. Again, this strain exhibited superior erythritol production, establishing this organism as an erythritol platform strain in a bioreactor setting. Here, the production of erythritol achieved 78 g/L (Fig. 5b), and *Q*_ERY_ and *Y*_ERY_ were significantly enhanced to 1.08 g/L/h and 0.52 g/g, respectively (Fig. 6). Moreover, this engineered strain exhibited reduced CA and biomass production to enable heightened flux towards erythritol synthesis (Fig. 5b; Table 4). This result was puzzling since the single overexpression of *GUT2* leads to enhanced CA synthesis. During *GUT2* expression, NAD^+^ is reduced to NADH, which in cells is regenerated during oxidative phosphorylation. Electrons released during NADH, the reoxidation flow through the electron-transport chain to generate a proton gradient across the membrane. Next, these protons flow through ATP synthase to generate ATP from ADP and inorganic phosphate. Therefore, excess NADH leads to enhanced intracellular ATP concentration. Moreover, it is known that the TCA cycle is strongly inhibited by excessive ATP [32]. However, in *Aspergillus niger* in the CA high-yield strains, there is an alternative pathway for NADH regeneration without ATP synthesis [33]. A too-high level of intracellular NADH is undesirable for cells because of the increasing ATP concentration, therefore *A. niger* cells to undergo an alternative regeneration of NADH without ATP synthesis. As a consequence, the concentration of intracellular ATP decreases and the TCA cycle becomes highly active, which results in elevated CA production. Therefore, we suggest that a similar mechanism might occur in *Y. lipolytica* during *GUT2* overexpression at high osmotic pressure. This situation might explain why the coexpression of *GUT1* and *GUT2* results in enhanced erythritol synthesis and reduced CA titer. In this strain, the overexpression of *GUT1* requires an enhanced level of ATP. Therefore, excess NADH (caused by *GUT2* overexpression) is used for the ATP synthesis required by glycerol kinase. Consequently, a high level of intracellular ATP inhibits the TCA cycle. However, this hypothesis requires further studies pertaining to CA production in *Y. lipolytica*.

### Citric acid production of the *GUT1* and *GUT2* transformants at pH 3.0

Finally, we also tested CA production by the modified strains at pH 3.0. Prior to this study, it was generally accepted that *Y. lipolytica* was unable to produce large quantities of CA at low pH. However, during the course of prior experiments in flasks, we observed that *GUT1*- and/or *GUT2*-overexpressing strains produced large quantities of CA at low pH. The possibility of producing metabolites at low pH (3.0) is of great importance in industrial applications because it avoids bacterial contamination, reduces production costs and enables non-septic conditions. In the fuel ethanol industry for example, sulfuric acid washing step is applied to decrease bacterial contamination. However, this process increases production costs [34]. Therefore, we sought to cultivate all engineered strains in a bioreactor to verify this phenomenon at the enhanced scale.

The strain A101 was used as a control (Table 5), and it consumed 150 g/L of glycerol within 96 h of cultivation. Consistent with a previous study [15], the strain A101 produced only 4.4 g/L of CA, achieving *Q*_CA_ 0.05 g/L/h and *Y*_CA_ 0.03 g/g (Table 2, Fig. 6). However, the culture quickly grew to a biomass concentration that exceeded 22 g/L within 48 h, and the biomass accumulation increased only minimally afterwards. On the other hand, the strain AJD pADUTGut1 assimilated 150 g/L of glycerol within 48 h of fermentation. Over the course of the process, 60.4 g/L of CA was produced (Fig. 5), resulting in *Q*_CA_ 0.63 g/L/h and *Y*_CA_ 0.40 g/g (Table 2), nearly a 13-fold increase over the control bioreactor. Interestingly, erythritol was first produced. However, during the cultivation, all the side metabolites, including erythritol, were fluxed toward CA synthesis (Table 5). Strikingly, a similar effect was observed during *GUT2* overexpression. Here, the titer of CA was slightly lower, 54.3 g/L, but the productivity attained value of 0.57 g/L/h, more than 11-fold that of the control. The most dramatic increase in CA production at low pH was observed during *GUT1/GUT2* overexpression. For this strain, the glycerol was fully assimilated over the course of the 72-h fermentation, and final CA value that was achieved was 64 g/L (Fig. 5; Table 5). Therefore, the titer increased 14.5-fold over that of the control. Moreover, the *Q*_CA_ (0.69 g/L/h) and *Y*_CA_ (0.43 g/g) increased 14-fold relative to the control bioreactor (Table 2, Fig. 6). This study presents the highest reported yield and productivity of CA at low pH to date. Interestingly, we observed a higher level of isocitric acid production in the engineered strains in comparison to the wild type. The level of isocitric acid (ICA) for the engineered strains oscillated 10–12 g/L, whereas for the wild type it achieved 0.35 g/L. This content is much higher, that in other studies proceeded for *Y. lipolytica* strains [14, 29], however in our study, strains were grown at low pH, what might have an influence on the isocitric acid synthesis. It is worth noting that under these conditions all of the modified strains first produced erythritol. However, after rapid glycerol depletion the carbon flux was forced toward CA synthesis. This effect was not observed in the medium with high osmotic pressure; this difference accordingly demonstrates the significance of environmental conditions on genetic targets for metabolic engineering.

**Table 5:** Citric acid and by-product synthesis by various *Y. lipolytica* strains in CA Fermentation Medium (pH 3.0)

Strain	Time (h)	Biomass (g/L)	Glycerol (g/L)	Erythritol (g/L)	Arabitol (g/L)	Mannitol (g/L)	Citric acid (g/L)	α-Ketoglutaric acid (g/L)
A101	24	12.1 ± 4.4	127.6 ± 12.1	3.9 ± 1.8	0.5 ± 0.2	1.5 ± 0.6	0	1.8 ± 0.8
	48	22.8 ± 3.2	68.1 ± 12.1	14.0 ± 3.0	1.7 ± 0.6	12.2 ± 1.3	0.9 ± 1.2	2.0 ± 0.1
	72	26.0 ± 4.8	16.3 ± 3.1	15.4 ± 3.9	2.7 ± 1.1	27.7 ± 7.6	4.4 ± 0.3	0.8 ± 0.1
	96	26.2 ± 2.8	0	6.2 ± 2.8	2.7 ± 0.1	26.1 ± 5.9	4.4 ± 1.8	0.8 ± 0.1
AJD pADUTGut1	24	19.1 ± 1.1	101.4 ± 0.8	11.1 ± 2.0	2.6 ± 1.3	2.4 ± 1.1	2.0 ± 1.5	0.4 ± 0.1
	48	24.0 ± 2.5	0	41.4 ± 0.1	6.8 ± 2.3	18.4 ± 2.3	33.0 ± 3.4	0.6 ± 0.8
	72	24.0 ± .0.2	0	10.3 ± 2.4	5.8 ± 0.8	18.4 ± 1.6	47.2 ± 1.4	0
	96	21.7 ± 1.8	0	0.7 ± 0.2	6.2 ± 1.0	18.3 ± 3.3	60.4 ± 6.8	0
AJD pADUTGut2	24	15.2 ± 2.1	98.4 ± 4.9	9.8 ± 0.7	1.2 ± 0.9	1.5 ± 0.1	1.2 ± 0.6	1.3 ± 0.1
	48	25.4 ± 3.9	14.0 ± 3.1	34.5 ± 3.9	3.1 ± 2.9	14.6 ± 2.4	29.8 ± 2.8	0
	72	24.6 ± 2.1	0	2.5 ± 1.5	3.1 ± 2.9	17.9 ± 5.6	53.6 ± 8.0	0
	96	24.1 ± 1.0	0	0	2.0 ± 1.4	13.7 ± 2.5	54.3 ± 4.6	0
AJD pADUTGut1/2	24	16.5 ± 0.5	90.6 ± 5.9	9.8 ± 0.2	1.1 ± 0.5	1.7 ± 0.2	1.1 ± 0.4	1.3 ± 0.1
	48	27.6 ± 1.0	19.4 ± 6.4	33.4 ± 5.9	3.3 ± 1.8	15.1 ± 1.3	29.8 ± 3.2	0.9 ± 0.6
	72	25.1 ± 0.4	0	4.2 ± 2.9	3.4 ± 1.3	20.2 ± 2.0	60.1 ± 1.3	0
	96	23.4 ± 1.0	0	0	3.0 ± 0.5	16.1 ± 0.7	63.9 ± 1.3	0

With further modification coupled with process modification, *Y. lipolytica* with enhanced glycerol assimilation has the potential to yield promising breakthroughs in efficient, rapid metabolite synthesis from low-cost substrates. With this platform, many natural and artificial metabolic pathways may be enhanced, thereby leading to the efficient synthesis of desirable products on an industrial scale.

## Conclusions

We have demonstrated metabolic engineering for the synthesis of value-added products from the low-cost substrate glycerol via the yeast *Y. lipolytica*. Over the course of this study, erythritol production increased over 35 % and the production of CA at low pH increased from 4.4 to 64 g/L (i.e., an improvement of 14.5-fold). Moreover, the process parameters were significantly enhanced; this platform demonstrated erythritol productivity from glycerol of 1.08 g/L/h, and a CA productivity at pH 3.0 of *Q*_CA_ 0.69 g/L/h and *Y*_CA_ 0.43 g/g. These values represent one of the highest reported CA titers at low pH and enhanced erythritol productivity to date in an industrially relevant organism. Moreover, these values were obtained using an inexpensive medium in which the single carbon source was glycerol; this situation is beneficial for downstream processing. The production of erythritol and CA is more efficient because of the short fermentation time. This shortened fermentation time should be valuable for industrial applications. This study accordingly presents a promising starting platform for further modifications of a broad range of value-added products related to biosynthesis from glycerol.

### Additional file

**Additional file 1: Fig.** **S1.** Copy number of *GUT1* and *GUT2* genes in the engineered *Y. lipolytica* strains. Wild type strain A101 was used as a control.

## Abbreviations

CA: citric acid
GK: glycerol kinase
GDH: glycerol-3-P dehydrogenase
*GUT1*: gene *YALI0F00484g* encoding glycerol kinase
*GUT2*: gene *YALI0B02948g* encoding glycerol-3-P dehydrogenase
ERY: erythritol
*Q*: productivity
*Y*: yield
