# Supplementary figures and images for: A novel strain of Yarrowia lipolytica as a platform for value-added product synthesis from glycerol
Source: Biotechnol Biofuels. 2016 Aug 30;9:180. doi: 10.1186/s13068-016-0593-z (PMC5009880; doi:10.1186/s13068-016-0593-z)

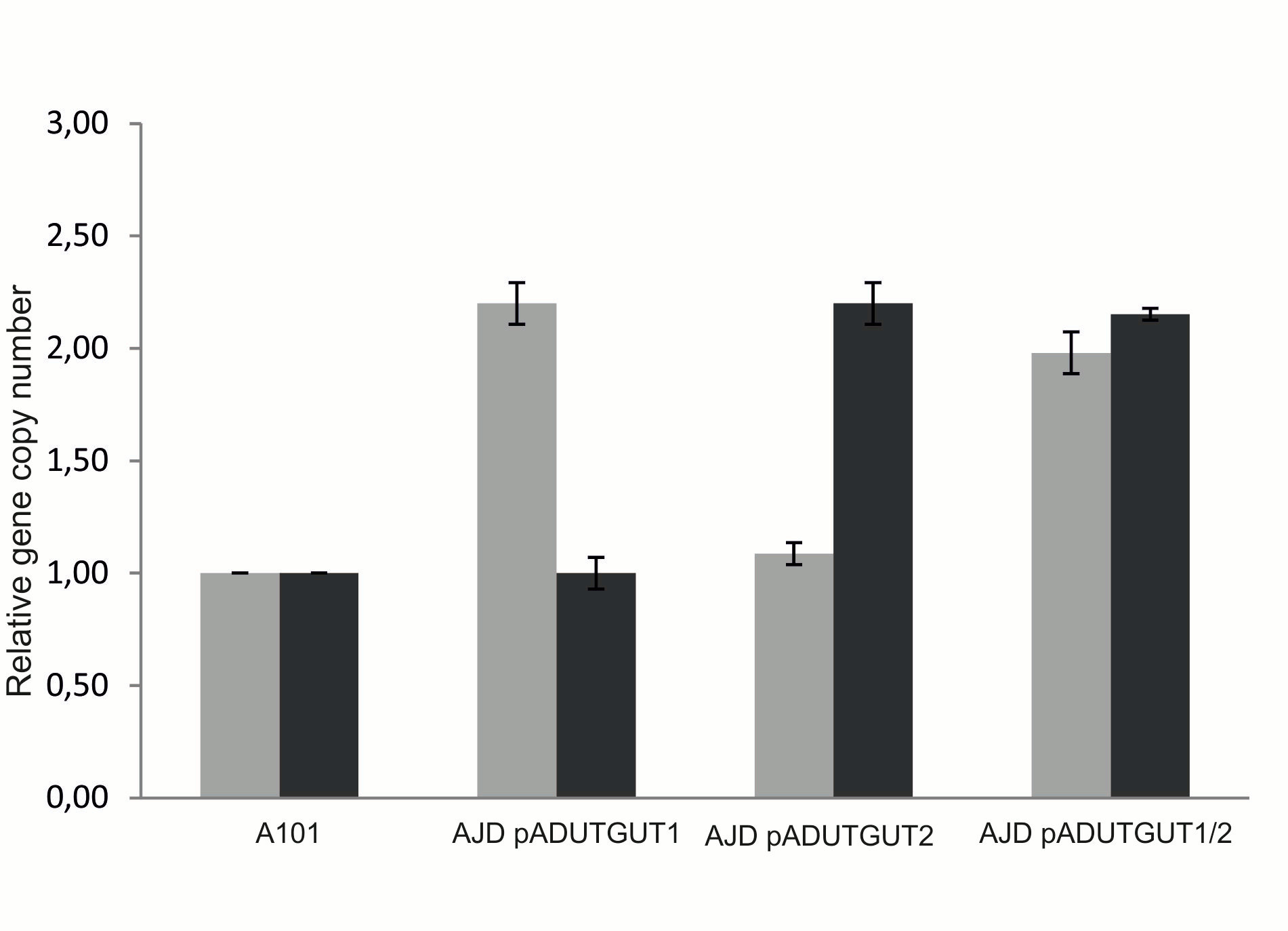

Supplement: Supplementary file 1 — Additional file 1: Fig. S1. Copy number of GUT1 and GUT2 genes in the engineered Y. lipolytica strains. Wild type strain A101 was used as a control. [file 13068_2016_593_MOESM1_ESM.jpg]
